# Supplementary material for: Feeding behavior and activity of Phlebotomus pedifer and potential reservoir hosts of Leishmania aethiopica in southwestern Ethiopia
Source: PLoS Negl Trop Dis. 2020 Mar 20;14(3):e0007947. doi: 10.1371/journal.pntd.0007947 (PMC7112221; doi:10.1371/journal.pntd.0007947)
Supplement: S1 Table — (PDF) [file pntd.0007947.s001.pdf]

Habitat	Comparison	Estimate	Standard error	p-value
Overall	Human-hyrax	1.555	0.352	<0.001
	Human-livestock	1.070	0.319	0.007
	Human-rodent	2.384	0.511	<0.001
	Human-other	2.339	0.466	<0.001
	Hyrax-livestock	-0.485	0.421	0.778
	Hyrax-rodent	0.829	0.580	0.609
	Hyrax-other	-0.783	0.541	0.596
	Livestock-rodent	1.314	0.560	0.131
	Livestock-other	1.269	0.520	0.105
	Other-rodent	0.045	0.655	1.000
Indoors	Human-hyrax	3.344	0.584	<0.001*
	Human-livestock	1.041	0.211	<0.001*
	Human-rodent	2.497	0.391	<0.001*
	Human-other	3.056	0.509	<0.001*
	Hyrax-livestock	-2.303	0.602	0.001*
	Hyrax-rodent	-0.847	0.686	0.731
	Hyrax-other	-0.288	0.759	0.996
	Livestock-rodent	1.455	0.417	0.005*
	Livestock-other	2.015	0.529	0.001*
	Other-rodent	-0.560	0.623	0.898
Outdoors	Human-hyrax	1.254	0.802	0.522
	Human-livestock	0.154	0.556	0.999
	Human-rodent	1.954	1.069	0.362
	Human-other	1.254	0.802	0.522
	Hyrax-livestock	-1.109	0.816	0.663
	Hyrax-rodent	-0.693	1.225	0.980
	Hyrax-other	0.000	1.000	1.000
	Livestock-rodent	1.799	1.080	0.460
	Livestock-other	1.109	0.816	0.663
	Other-rodent	0.693	1.225	0.980
Cave	Human-hyrax	0.069	0.372	0.999
	Human-livestock	2.015	0.753	0.057
	Human-rodent	2.708	1.033	0.066
	Human-other	2.708	1.033	0.066
	Hyrax-livestock	1.946	0.756	0.075
	Hyrax-rodent	2.639	1.035	0.080
	Hyrax-other	2.639	1.035	0.080
	Livestock-rodent	0.693	1.225	0.979
	Livestock-other	0.693	1.225	0.979
	Rodent-other	0.000	1.414	1.000

** = statistically significant*
