# Supplementary material for: Feeding behavior and activity of Phlebotomus pedifer and potential reservoir hosts of Leishmania aethiopica in southwestern Ethiopia
Source: PLoS Negl Trop Dis. 2020 Mar 20;14(3):e0007947. doi: 10.1371/journal.pntd.0007947 (PMC7112221; doi:10.1371/journal.pntd.0007947)
Supplement: S2 Table — (PDF) [file pntd.0007947.s002.pdf]

Sex	Comparison	Estimate	Standard error	p-value
Indoors	18h-20h - 20h-22h	-0.606	0.639	0.999
	18h-20h - 22h-24h	-0.414	0.643	1.000
	18h-20h - 24h-2h	-1.352	0.642	0.619
	18h-20h - 2h-4h	-0.001	0.661	1.000
	18h-20h - 4h-6h	1.133	0.785	0.955
	20h-22h - 22h-24h	0.193	0.620	1.000
	20h-22h - 24h-2h	-0.746	0.616	0.988
	20h-22h - 2h-4h	0.605	0.639	0.999
	20h-22h - 4h-6h	1.739	0.786	0.501
	22h-24h - 24h-2h	-0.938	0.622	0.639
	22h-24h - 2h-4h	0.413	0.643	1.000
	22h-24h - 4h-6h	1.547	0.771	0.689
	24h-2h - 2h-4h	1.351	0.642	0.620
	24h-2h - 4h-6h	2.485	0.772	0.058
	2h-4h - 4h-6h	1.133	0.785	0.955
Outdoors	18h-20h - 20h-22h	-0.548	0.745	0.999
	18h-20h - 22h-24h	-0.777	0.732	0.996
	18h-20h - 24h-2h	-0.652	0.797	0.999
	18h-20h - 2h-4h	0.575	0.956	1.000
	18h-20h - 4h-6h	1.359	1.185	0.993
	20h-22h - 22h-24h	-0.229	0.676	1.000
	20h-22h - 24h-2h	-0.104	0.748	1.000
	20h-22h - 2h-4h	1.123	0.916	0.987
	20h-22h - 4h-6h	1.907	1.154	0.889
	22h-24h - 24h-2h	0.125	0.735	1.000
	22h-24h - 2h-4h	1.353	0.906	0.943
	22h-24h - 4h-6h	2.137	1.146	0.782
	24h-2h - 2h-4h	1.227	0.951	0.981
	24h-2h - 4h-6h	2.011	1.182	0.868
	2h-4h - 4h-6h	0.784	1.293	1.000
