# Supplementary material for: Feeding behavior and activity of Phlebotomus pedifer and potential reservoir hosts of Leishmania aethiopica in southwestern Ethiopia
Source: PLoS Negl Trop Dis. 2020 Mar 20;14(3):e0007947. doi: 10.1371/journal.pntd.0007947 (PMC7112221; doi:10.1371/journal.pntd.0007947)
Supplement: S3 Table — (PDF) [file pntd.0007947.s003.pdf]

| Sex | Comparison | Estimate | Standard error | p-value |
|---|---|---|---|---|
| Female | 19h-20h - 20h-21h | -0.542 | 0.138 | 0.005* |
| | 19h-20h - 21h-22h | -0.639 | 0.149 | 0.001* |
| | 19h-20h - 22h-23h | -0.668 | 0.141 | < 0.001* |
| | 19h-20h - 23h-24h | -0.588 | 0.144 | 0.003* |
| | 19h-20h - 24h-01h | -0.353 | 0.167 | 0.618 |
| | 20h-21h - 21h-22h | -0.098 | 0.124 | 1.000 |
| | 20h-21h - 22h-23h | -0.126 | 0.115 | 0.995 |
| | 20h-21h - 23h-24h | -0.047 | 0.119 | 1.000 |
| | 20h-21h - 24h-01h | 0.189 | 0.145 | 0.980 |
| | 21h-22h - 22h-23h | -0.029 | 0.127 | 1.000 |
| | 21h-22h - 23h-24h | 0.051 | 0.132 | 1.000 |
| | 21h-22h - 24h-01h | 0.286 | 0.150 | 0.758 |
| | 22h-23h - 23h-24h | 0.079 | 0.123 | 1.000 |
| | 22h-23h - 24h-01h | 0.315 | 0.149 | 0.618 |
| | 23h-24h - 24h-01h | 0.236 | 0.154 | 0.932 |
| Male | 19h-20h - 20h-21h | -0.657 | 0.362 | 0.809 |
| | 19h-20h - 21h-22h | -0.876 | 0.373 | 0.442 |
| | 19h-20h - 22h-23h | -0.580 | 0.379 | 0.933 |
| | 19h-20h - 23h-24h | -0.855 | 0.364 | 0.443 |
| | 19h-20h - 24h-01h | -0.351 | 0.438 | 1.000 |
| | 20h-21h - 21h-22h | -0.219 | 0.297 | 1.000 |
| | 20h-21h - 22h-23h | 0.077 | 0.304 | 1.000 |
| | 20h-21h - 23h-24h | -0.199 | 0.286 | 1.000 |
| | 20h-21h - 24h-01h | 0.306 | 0.375 | 1.000 |
| | 21h-22h - 22h-23h | 0.296 | 0.318 | 0.999 |
| | 21h-22h - 23h-24h | 0.021 | 0.301 | 1.000 |
| | 21h-22h - 24h-01h | 0.505 | 0.384 | 0.970 |
| | 22h-23h - 23h-24h | -0.276 | 0.308 | 0.999 |
| | 22h-23h - 24h-01h | 0.229 | 0.584 | 1.000 |
| | 23h-24h - 24h-01h | 0.505 | 0.079 | 0.975 |
