# Supplementary material for: Feeding behavior and activity of Phlebotomus pedifer and potential reservoir hosts of Leishmania aethiopica in southwestern Ethiopia
Source: PLoS Negl Trop Dis. 2020 Mar 20;14(3):e0007947. doi: 10.1371/journal.pntd.0007947 (PMC7112221; doi:10.1371/journal.pntd.0007947)
Supplement: S4 Table — For each of the eight iterations of the experiment, the number (%) of sand flies that were used for the experiment, that eventually took a blood meal and which host they were found to feed on are presented. (PDF) [file pntd.0007947.s004.pdf]

Experiment number	Total amount of female sand flies	Total amount of blood fed female sand flies (%)	Total amount of sand flies fed on hyrax (%)	Total amount of sand flies fed on human (%)
**1**	95	5 (5.3%)	1 (20.0%)	4 (80.0%)
**2**	118	13 (11.0%)	9 (69.2%)	4 (30.8%)
**3**	65	11 (16.9%)	6 (54.6%)	5 (45.5%)
**4**	80	2 (2.5%)	0 (0.0%)	2 (100.0%)
**5**	34	4 (11.8%)	2 (50.0%)	2 (50.0%)
**6**	108	6 (5.6%)	5 (83.3%)	1 (16.7%)
**7**	106	18 (16.0%)	13 (72.2%)	5 (27.8%)
**8**	110	6 (5.5%)	4 (66.7%)	2 (33.3%)
**Total**	716	65 (9.0%)	40 (61.5%)	25 (38.5%)
