# Supplementary material for: Feeding behavior and activity of Phlebotomus pedifer and potential reservoir hosts of Leishmania aethiopica in southwestern Ethiopia
Source: PLoS Negl Trop Dis. 2020 Mar 20;14(3):e0007947. doi: 10.1371/journal.pntd.0007947 (PMC7112221; doi:10.1371/journal.pntd.0007947)
Supplement: Attachment — Submitted filename: Review.docx [file pntd.0007947.s009.docx]

**Response to reviewers**

Author’s responses to comments in blue

Reviewer's Responses to Questions

1. Methods

-Are the objectives of the study clearly articulated with a clear testable hypothesis stated?

-Is the study design appropriate to address the stated objectives?

-Is the population clearly described and appropriate for the hypothesis being tested?

-Is the sample size sufficient to ensure adequate power to address the hypothesis being tested?

-Were correct statistical analysis used to support conclusions?

-Are there concerns about ethical or regulatory requirements being met?

**Reviewer #1**: Some experiments need more detailed description, see my Summary and general comments.

Experiments have been explained with more detail as the reviewer suggested (see more below).

**Reviewer #2**: The study is well designed and methods fully and clearly described. The only point at issue is the fact that sand fly collection for bloodmeal analysis was not performed equally in three different habitats: CDC light traps and sticky traps were used indoors and in caves while outdoors, only sticky traps were used. In addition, collections in caves were performed in different months than collections indoors and outdoors. This heterogeneity is understandable but authors must interpret the results appropriately (more below).

The trapping methods and months indeed varied over the three different habitats. We tried to make this clear in our results section now to avoid misinterpretation of the results (see more below).

**Reviewer #3**: The objectives of the study are clearly articulated with the clear hypothesis. The study design is appropriate to the objectives, even if some additional procedure should be applied (eg *Leishmania sp* identification by PCR-RFLP analysis). Population and sample size are appropriate and adequate for the hypothesis tested. Correct statistical analysis support the conclusions and ethical requirements are being met.

Previous research in Ochollo has shown that *L. aethiopica* is the only parasite species causing CL in the area (identified from infected humans, sand flies and hyraxes). However, since the sample size was small, we identified the *Leishmania* species of the five kDNA positive sand flies and one goat nose biopsy. In contrast to the reviewer’s suggestion to conduct a PCR-RFLP analysis, we identified the *Leishmania* species by amplification of the ITS-1 gene followed by Sanger sequencing, as described in our previous paper (Pareyn et al. 2019, PLOS NTD). All kDNA positive blood fed sand flies carried *L. aethiopica* parasites. The *Leishmania* species of the kDNA positive goat nose biopsy sample could not be determined, because the parasitemia was probably to low (kDNA real-time PCR: Ct-value 28).

We added the protocol of the additional analysis and according results to the manuscript.

--------------------

2. Results

-Does the analysis presented match the analysis plan?

-Are the results clearly and completely presented?

-Are the figures (Tables, Images) of sufficient quality for clarity?

**Reviewer #1:**

Results are clearly and completely presented but I am missing data about temperature and relative humidity during the experiments on sand fly activity, for more details see Summary and general comments.

See response to this comment in “Summary and general comments”.

**Reviewer #2:**

Results are completely presented and all the figures have sufficient quality. However, the summary evaluation of blood meal sources (lines 289-294) is biased by higher representation of the indoor sample than both outdoor and cave samples. I suggest including the information that 129 from180 sand files came from indoor captures to the legend of the Table 1.

We agree that the data presented in Table 1 can be misinterpreted because most sand flies come from indoors. As suggested, we added this information to the legend of the table, so that the results can correctly be interpreted.

**Reviewer #3:**

The analysis matches to the analysis plan. Due to the fact that cutaneous leishmaniasis (CL) in Ethiopia are caused prevalently by *L. aethiopica* but also by *L. tropica* and *L.major* it is important that the AA identify *Leishmania* at species level and not simply to genus level, even simply by PCR-RFLP procedure. Otherwise the *Leishmania* molecular analysis remains incomplete. The results are clearly presented as the quality and clarity of Figures and Tables.

As described above (Methods), we now identified the *Leishmania* species based on ITS-1 sequencing. We demonstrate in the results section that all infected blood fed sand flies were *L. aethiopica* and that the positive goat nose biopsy sample could not be determined to species level.

--------------------

3. Conclusions

-Are the conclusions supported by the data presented?

-Are the limitations of analysis clearly described?

-Do the authors discuss how these data can be helpful to advance our understanding of the topic under study?

-Is public health relevance addressed?

**Reviewer #1:** Conclusions are the weakest part of the manuscript but could be easily corrected, see Summary and general comments.

We appreciated the reviewer’s concern about the conclusions. Therefore, we improved our paper by adjusting the conclusions as both reviewer 1 and 2 suggested, both in the abstract and discussion of the manuscript (see details below).

**Reviewer #2:** I do not agree with the conclusion that *Leishmania* transmission in SW Ethiopia is likely mainly anthroponotic (see the general comments and comments to discussion).

We changed the conclusion, stating that “besides zoonotic transmission, there is likely also human-to-human transmission” as both reviewer 1 and 2 suggested (see more below).

**Reviewer #3:** All the aspects of the conclusions are clearly supported and discussed, included the limitations of the analysis. The authors describe well the helpful to advance the topic under study. It is clear a paper of public health relevance in terms of surveillance and control of cutaneous leishmaniasis in Ethiopia.

--------------------

Editorial and Data Presentation Modifications?

Use  this section for editorial suggestions as well as relatively minor modifications of existing data that would enhance clarity. If the only modifications needed are minor and/or editorial, you may wish to recommend “Minor Revision” or “Accept”.

**Reviewer #1:**

*Abstract*

- Line 23-24: authors did not establish but studied the role of livestock in CL transmission.

Corrected.

- Line 32: indicate if all 180 were P. pedifer females.

Additional information is provided now.

- Line 38:  “amount” . Do you mean “number” or “proportion”?

The number of sand flies was included in the analysis. “Amount” was changed by “number” in the manuscript.

- Line 41-42: please correct the conclusions: 1) use more precise explanation about proposed transmission cycle (e.g. ..very likely, also anthroponotic transmission occurs in Ochollo”), see also my general comment. 2) Livestock was proposed as a potential reservoir for VL but never for CL, therefore the sentence needs to be corrected also in this aspect.

1) We agree with the reviewer that the conclusions that were made regarding the anthroponotic transmission need to be stated more carefully. However, we do think it would be correct to say that our findings represent southwestern Ethiopia instead of just Ochollo as the vector and ecology in the surrounding settings are very similar. We improved the conclusion to: “In contrast to earlier suggestions of exclusive zoonotic *Leishmania* transmission, we indicate that CL transmission is very likely also anthroponotic in southwestern Ethiopia.”
2) We appreciate the reviewers’ concern for misinterpretation of the sentence the way it was formulated before. Therefore, we adjusted this part of the conclusion to “Livestock does not play a role in CL transmission”.

*Background*

- Line 73-74: Mutinga and Odhiambo demonstrated susceptibility of *P. pedifer* to *L. aethiopica.* They did not study vectorial capacity. Moreover, susceptibility only may imply/suggest the efficient vector. Please, change the sentence accordingly.

We agree with the reviewer that the sentence requires reformulation. We corrected the sentence to: “A study in Kenya found a high susceptibility of *P. pedifer* to *L. aethiopica* when feeding on active human CL lesions, implying that it could be an efficient vector.”

In the discussion section of the full-text, this was already correctly formulated.

*Materials and Methods*

- Line 125-126: location of caves should be specified more. Are they really in the village? How far is from the caves to the nearest houses?

We added additional information about the caves where sampling was done in the methods section: “Caves were located within the village, in a range of maximum 300 meters from human dwellings.”.

- Line 221: explain better the position of the human volunteer during the experiment. How the human volunteer was separated from the opposite cage with the hyrax?

Additional information was added to the methods section: “Human volunteers were lying down in a horizontal position with their head nearby the cage for CO_2_ attractiveness towards the sand flies. The distance between the two hosts was three meters.” No further considerations were taken to separate the two hosts from each other.

- In the revised version please correct the format, in some pages (e.g. lines 137, 159 and elsewhere) the subtitles were merged with the text (please place the text on the new line).

The in-line headings are described in the journal’s submission guidelines.

*Results*

- Line 286: I suggest correction “..sand fly species of subgenus *Larroussius*”

We agree with the reviewer and adapted the sentence as requested.

*Discussion*

- Line 467: I suggest introducing somehow the paragraph, e.g. by “In conclusion….”.

We added this in the discussion section as requested by the reviewer.

*References*

- Please check names of the authors again, you give three references by Ashford but only one of them is correct (Ashford RW, not Ashford W, as written in refs. 12 and 38).

**Corrected.**

**Reviewer #2:**

*Abstract*

- Lines 32-35: Second and third sentences should be changed to: Humans were the predominant blood meal source indoors (65.9%, P <0.001) while no significant differences in blood meal sources were determined outdoors and in caves. In caves, hyraxes were represented in blood meals equally as humans (45.5% and 42.4 %, respectively), but the host choice experiment revealed…

We agree with the reviewer that the way the results were described in the previous version were biased because most sand flies were collected indoors, where humans are obviously the most accessible host. Hence, we changed the result in the abstract exactly as the reviewer suggested. Additionally, we emphasize on the fact that there were no significant differences among the blood meal sources of the cave and outdoor habitats in the results section of the improved manuscript.

- Line 42: please change the conclusion that *Leishmania* transmission in SW Ethiopia is likely mainly anthroponotic (see the general comments and comments to discussion).

This conclusion has been rephrased in the abstract as both reviewer 1 and 2 have suggested: “In contrast to earlier suggestions of exclusive zoonotic *Leishmania* transmission, we indicate that CL transmission is very likely also anthroponotic in southwestern Ethiopia.”

*Author summary*

- Lines 51-52: the sentence “humans are likely the main source of the infection “should be changed to “humans are an important source of the infection”

Corrected as suggested.

*Methods*

- Line 137 Blood isolation from the blood fed specimens („from“ is missing)

Corrected.

- Line 215 – citation of the original description of the method is missing (I suppose Sadlova et al.2003, <https://eur01.safelinks.protection.outlook.com/?url=https%3A%2F%2Fdoi.org%2F10.1046%2Fj.1365-2915.2003.00434.x&amp;data=02%7C01%7Cmyrthe.pareyn%40uantwerpen.be%7C4949034e2e3741aca7b508d78c63f02d%7C792e08fb2d544a8eaf72202548136ef6%7C0%7C0%7C637132233175334895&amp;sdata=Ttp5BddnMguZjzB8Tvc4bIp0GoulglgMCiatNsKjggM%3D&amp;reserved=0.>)

We added this reference to the manuscript.

*Discussion*

- Lines 370-371. Sentence „ On the contrary, only 10.6% of the sand fly blood meals were derived from hyraxes and none of these sand flies were found kDNA positive should be followed by the sentence „However, the low representation of hyraxes in the blood meals may be biased by high prevalence of specimens captured indoor in the analysed material.“ and also the following sentences adapted according to this fact.

As mentioned before in the results section, this part indeed needed re-interpretation to avoid biased conclusions. In the revised version of the manuscript we describe: “Only 10.6% of the sand fly blood meals were derived from hyraxes, however, the low representation of hyraxes in the blood meals may be biased by the higher proportion of specimens captured indoors in the analysed material. None of the sand flies that fed on hyraxes were found kDNA positive, which is an interesting result because…”

- Lines 374-375 Instead of ”..this study demonstrates that there is very likely also anthroponotic transmission..” should be written “ ..this study suggests that human-to-human transmission may be involved ..”

We rephrased the sentence as the reviewer suggested.

- Line 467 I suggest to write „often feed on humans and, therefore, human-to-human transmission should be considered” instead of „mainly feed on humans and that there is likely also anthroponotic transmission”

We changed the sentence to: “This study shows that sand flies in Ochollo often feed on humans and, therefore, human-to-human transmission of *L. aethiopica* should be considered.”

**Reviewer #3:**

The paper of Pareyn et al 2019 (PLOS NTD) appears to play the role of prerequisite of the present survey and not simply as a reference.

Our previous research published in 2019 (reference 13) described the seasonality of (infected) sand flies, the habitat of (infected) sand flies and the role of hyraxes and rodents in CL transmission. These findings and the current results can be used as a guidance for disease control. However, we think that there is no need to emphasize more on our first paper (13), as the results of the previous paper did not lead to the research questions of this study.

--------------------

Summary and General Comments

Use  this section to provide overall comments, discuss strengths/weaknesses of the study, novelty, significance, general execution and scholarship. You may also include additional comments for the author, including concerns about dual publication, research ethics, or publication ethics. If requesting major revision, please articulate the new experiments that are needed.

**Reviewer #1:**

Authors studied activity and feeding preferences of *P. pedifer* in the well-known Ethiopian focus of cutaneous leishmaniasis caused by *L. aethiopica*. In addition, they search for *Leishmania* presence in livestock by molecular methods. Results are interesting and important; however, their interpretation needs some corrections and some more detailed description of the experiments would be useful:

1. I appreciate that the work was done in natural conditions, but it would be useful to describe some experiments in more details, see my specific comments. More importantly, I am missing data about ambient temperature and relative humidity during the study on biting patterns and CDC light trap captures (Lines 331-340). Sand fly activity is highly dependent on these parameters and I expect that some differences observed might be explained by differences in humidity and temperature. Did you use data loggers and could you add these data?

Additional information regarding the caves that were used for sand fly collections and set-up of the host preference experiment were added to the methods section as the reviewer requested (see above). We appreciate the reviewers suggestion that data on temperature and humidity would complement well with our results, because the activity could indeed depend on the temperature. Unfortunately, we did not record these data during the sampling time and the nearest weather station is situated too far from Ochollo to provide meaningful data for our setting.

2. The study shows that livestock has no role in CL transmission but serve as a blood source. This is just a confirmation of other previous studies on CL. Therefore, in the Abstract two sentences in Background (lines 23-24) and conclusion (lines 41-42) should be corrected. Same is required for the sentence in the Background on lines 88-89. Bovines are frequently bitten by sand flies in many countries, e.g. in East Mediterranean or Magreb, but they do not serve as reservoirs there. Please, make all these corrections.

We agree with the reviewer regarding the word choice we used in the abstract and introduction regarding the role of livestock in CL transmission. The sentences were rephrased. In the introduction, however, we only state that it has been found that CL vectors from Ethiopia and Kenya are biting livestock, so that therefore we could try to investigate their role in transmission. We think it would not be very useful to make comparisons with other countries, as the vector, parasites and ecological settings are not comparable with Ethiopia. We slightly adapted the statement and describe now that because bovines often get bitten by CL vectors in Ethiopia, their role in transmission needs further investigation.

3. Similarly, in Discussion (lines 358-9) there is the sentence “We gathered novel insight in …..the role of livestock in transmission of CL in southwestern Ethiopia, which can be used as a guidance in disease control programs”. What is the novel insight concerning the livestock? I did not find any. What do you mean by this general sentence? Does it mean that the livestock should be sprayed or not?

The sentence “we gathered novel insights in the biting behavior and activity of *P. pedifer* and the role of livestock in transmission of CL in southwestern Ethiopia, which can be used for guidance in disease control programs” more refers to the results we gathered regarding the sand fly behavior. We agree with the reviewer that the order of the sentence is confusing. We adapted the introductory sentence of the discussion to: “We gathered novel insights in the biting behavior and activity of *P. pedifer*, which can be used as a guidance in disease control programs; and studied the role of livestock in transmission of CL in southwestern Ethiopia.” Whether or not livestock could be used for control strategies (e.g. insecticide spraying) needs further investigation, before we can draw any conclusions. This is described in the discussion section.

4. Authors bring the evidence that many *P. pedifer* feed on humans, which are probably the most common and most easily accessible mammals in the area. This, however, does not mean that the disease is anthroponotic, it seems to me that there is a combination of anthroponotic and zoonotic transmission, as correctly mentioned by authors in Discussion (lines 374-5: “… that there is very likely also anthroponotic transmission….). Please, change the conclusion on lines 41-42 accordingly.

As described above, the conclusion of the abstract has been adapted as the reviewers suggested, stating that besides zoonotic transmission, there is probably also human-to-human transmission.

**Reviewer #2:**

The study is aimed at feeding preferences and biting activity of sand flies (*Phlebotomus pedifer*) in the CL foci in SW Ethiopia caused by *L. aethiopica*. In addition, *Leishmania* presence in livestock (goats and bovines) was studied. The method used is appropriate and the results are novel and interesting. Valuable is the description of the broad host spectrum of *P. pedifer* and detailed description of the temporal biting pattern of this sand fly species. However, the summary interpretation of the data and some conclusions need revision. Authors speculate about the anthroponotic transmission of *L. aethiopica* in Ochollo based on the fact that majority (59%) of sand flies fed on humans and only 10% on hyraxes. However, in their samples, 129 flies were captured indoors in comparison with only 18 captured outdoor and 33 in caves. Importantly, representation of human blood was not significantly higher in outdoor and cave samples and in direct preference test, *P. pedifer* preferred hyraxes over humans. My specific suggestions are written in comments to respective parts of the manuscript.

Conclusions in the abstract and discussion have been adapted. We now state that besides zoonotic transmission, there is probably also human-to-human transmission. Furthermore, we added to the legend of Table 1 of the blood meal sources that most of the blood fed sand flies were derived from indoors to avoid misinterpretation of the results. Also in the results section of the revised manuscript, we emphasize on the fact that there is no difference in the blood meal sources outdoors and in caves.

**Reviewer #3:**

The manuscript on the “Feeding behavior and activity of *Phlebotomus pedifer* and potential reservoir hosts of *Leishmania aethiopica* in southwestern Ethiopia” is interesting and well structured. However, the survey, based on molecular approaches, suffers sometime of the no application of parasitological classical procedures. In addition the paper of Pareyn et al 2019 appears to play the role of prerequisite of the present survey and not simply as a reference. For example, rodents analysis was better evaluated in Pareyn et al as the Leishmania species identification.

Due to the fact that cutaneous leishmaniasis (CL) in Ethiopia are caused prevalently by *L. aethiopica* but also by *L. tropica* and *L. major* it is important that the AA identify *Leishmania* at species level and not simply to genus, even simply by PCR-RFLP procedure. Otherwise the *Leishmania* molecular analysis remains incomplete. Hyrax xenodiagnosis studies should be implemented to solve the *L. aethiopica* role of vector and reservoir hosts.

Since the Results the text appears not accurate, as the rest of the manuscript, and sometimes it is confused.

As the reviewer requested, we identified the *Leishmania* species that was found in the kDNA positive sand flies; only *L. aethiopica* DNA was found. Indeed, it would have been even more interesting to find live *Leishmania* parasites (culture), but 1) for the sand flies we needed intact blood fed specimens for the blood meal identification, so we could not use the samples for dissections and microscopy 2) for the bovines and goats it was our intention to do a primary screening to check if we could find at least some DNA in the tissue samples. If we would have found a high prevalence of *Leishmania* DNA in the livestock samples, then we could have tried to isolate live parasites, which is not the case in our study setting.

Specific comments

- Lines 23: AA should insert “Southern Ethiopia”, the geographic location of the area studied.

We added “in southwestern Ethiopia”.

- Line 46: Correct “is caused” in “is mainly caused”. Three Leishmania species (without consider L. donovani….) cause CL in Ethiopia.

We agree with the reviewer and added “mainly”.

- Line 50 : What intend the AA as “other potential hosts”? Reservoir Hosts? Please clarify.

We indeed meant other reservoir hosts. Up to now, only hyraxes and rodents have been investigated for their role in transmission. We changed it to “reservoir hosts” as the reviewer suggested.

- Line 72-81: Paper “13” is basic for the project planning. This should be a prerequisite in the Introduction.

We are not entirely sure what the reviewer means with this comment. As explained above, we do not think that our previous research led to the objectives of this study, hence we think there is no need to describe these previous results in more detail.

- Lines 139: The AA should specify the *Leishmania sp* infected mice. If it is *L. major*, as in Pareyn et al 2019, give species and ref, otherwise *Leishmania* species should be given complete of the strain WHO code.

The mice were not infected with *Leishmania*. Sand flies from a colony were fed on naive mouse blood. These blood fed sand flies were used as a positive control for the DNA extraction. If after the Cytochrome B PCR (band visible) and sequencing (BLAST analysis) the blood meal source of this sand fly appeared mouse blood, the assay was considered successful.

- Line 181-182: These 2 lines could be removed doing follow the appropriate reference from line 172 (ref 25 for RT-qPCR SL-RNA, ref 13 for qPCR kDNA).

We appreciate the reviewers concern, but we think it is clearer for the reader if the lines are kept in the manuscript. It is required in the beginning of the paragraph to understand why two different extractions are being done, but nucleic acid extraction is not described yet in the first part of the paragraph, so it would be confusing to first explain how to conduct the PCRs and then the nucleic acid isolation. However, we added the references (Eberhardt 2018 and Pareyn 2019) already in the first part of the paragraph as the reviewer suggests.

- Line 279: The AA should give the total of the collected sand flies from the 72 households. Total number could justify because sand flies were not identified by morphological keys.

A total of 11,488 sand flies were collected, of which 368 were found blood fed. We added this information to the revised version of the manuscript. Indeed, we did not identify all sand flies to species level, only the ones that we successfully identified the blood meal source from (as explained in the methods section).

- Line 293: The sand flies that fed on humans are 107 not 137. Please correct.

The reviewer is right. We corrected the mistake in the results section. Additionally, the percentage of infected human fed sand flies was incorrect in the discussion section, so this was also corrected from 3.6% to 4.7%.

- Line 369-370: Ref 14 concerns *L. aethiopica* but in Kenya.

It seemed indeed from the way the sentence was formulated that this study was carried out in Ochollo. We rephrased the sentence in the new version of the manuscript to clarify that it was carried out in Kenya: “… and a study in Kenya demonstrated that *L. aethiopica* transmission from a human CL lesion to *P. pedifer* is very efficient.”

- Lines 371-73: The comment is due by the structure of the work. In the present manuscript human role was one of the principal objectives, different structure was in Pareyn et al 2019.

In our previous study (Pareyn et al 2019) we found that there is a high infection prevalence among the hyraxes in Ochollo, hence they probably play an important role in transmission. However, if transmission of *L. aethiopica* from the hyrax to *P. pedifer* would be very efficient (as was shown for humans), we would expect that now at least some of the sand flies that fed on a hyrax would contain *Leishmania* DNA, which was not the case. Therefore, we suggest that a xenodiagnosis experiment should be carried out to determine their true role in transmission. This is a new finding from the current manuscript, so we think that it should be mentioned.

- Line 382: The AA should report ref 13 following “(2019)”.

As mentioned before, we think it is a bit odd to refer to our own study as Pareyn et al., since the first author is the same. Therefore, we prefer to refer to it as “our previous study”.

Many thanks to all three reviewers for their constructive comments and suggestions!

--------------------
