# Supplementary material for: Feeding behavior and activity of Phlebotomus pedifer and potential reservoir hosts of Leishmania aethiopica in southwestern Ethiopia
Source: PLoS Negl Trop Dis. 2020 Mar 20;14(3):e0007947. doi: 10.1371/journal.pntd.0007947 (PMC7112221; doi:10.1371/journal.pntd.0007947)
Supplement: Attachment — Submitted filename: Review2.docx [file pntd.0007947.s010.docx]

**Response to reviewers**

Author’s responses to comments in blue

Editor:
Please modify the text as required by the Reviewer 1's comment, with which I agree, copied below:

Authors significantly improved the manuscript and I agree with all but one correction they did. The term “anthroponosis” should be use only if humans are the sole reservoir hosts. Therefore, I think that at least the sentence in the abstract: “In contrast to earlier suggestions of exclusive zoonotic   Leishmania   transmission, we indicate that CL transmission is very likely also anthroponotic in southwestern Ethiopia.” should be changed to e.g. “In contrast to earlier suggestions of exclusive zoonotic   Leishmania   transmission, we propose that there is also human-human transmission of CL in southwestern Ethiopia.”

We agree with issue the editor and reviewer 1 raised and changed the sentence in the abstract as suggested.

No further comments were made by the reviewers.
